# The Prussian Siege of Paris

**Published:** 1871-10

**Authors:** 


The Prussian Siege of Paris.—The profession has been put in possession of a few particulars in regard to the bearings of the first or Prussian Siege of Paris, in hygiene and surgery, a paper by Dr. Gordon, C. B., on the subject having been read before the British Medical Association at Plymouth, a few weeks ago, and more recently published at length in the journal of that body. Dr. Gordon, alluding to the physique of the men who were drafted into the battalions formed in Paris, in the vain hope of resisting the approaching powerful enemy, remarks that they were in a great measure undeveloped lads, unsuited in bodily strength for the hardships of a campaign. The clothing supplied to them, although apparently of good quality, was insufficient in quantity; food, scarce almost from the commencement of the siege, was altogether insufficient in quantity long before the capitulation took place, while at the same time fuel became a thing almost unknown, and all this in a winter of unusual severity, so that the sufferings of the troops must at times have been very great. A new and unexpected question is raised in reference to preserved or tinned meats, the suitability of which as food is doubted by Dr. Gordon, whose remarks upon the subject almost seem to receive confirmation in the reports from the camps of the autumn manœuvres supplied to the daily press by correspondents. Dr. Gordon, aware of the importance of the question, writes cautiously in regard to it, but considering the suggestive bearing it has to long sea voyages and to future campaigns, it will it is hoped obtain further attention.

It would seem that the male nurses in the Paris hospitals were neither well-informed in regard to their duties, nor desirable attendants in other respects. There appeared to be some considerations however, which led Dr. Gordon, while writing in respectful terms of the female nurses, to suggest their unsuitableness for military hospitals, and he would accordingly have them replaced by well trained and respectable men.

The great mortality among the wounded is alluded to, and mortality to which several conditions seem to have conduced, such as improper and insufficient accommodations, previous hardships, cold, and insufficient food, and inadequate support in hospitals, *vin de Bordeaux* and *confiture* taking the place of beefsteak and porter. The question of conservative surgery is also glanced at, the impression evidently being that rules have yet to be laid down as to the conditions in which its practice should be adopted, as well as those for which its requirements are unsuited.—*The Doctor.*

